# Resilience’s impact on quality of life and post-traumatic growth in breast cancer patients during treatment

**DOI:** 10.1007/s12282-024-01594-2

**Published:** 2024-05-17

**Authors:** Songül Duran, Umut Varol, Özlem Tekir, Ahmet Hakan Soytürk

**Affiliations:** 1 Care of Elderly Program, İzmir Demokrasi University, Health Services Vocational College, İzmir, Türkiye; 2 Medical Oncology Clinic, İzmir Demokrasi University, İzmir Democracy University Buca Seyfi Demirsoy Training and Research Hospital, İzmir, Türkiye; 3 Faculty of Health Sciences, Department of Nursing, İzmir Demokrasi University, İzmir, Türkiye; 4 Kocaeli Dilova State Hospital, Kocaeli, Türkiye

**Keywords:** Breast cancer, Quality of life, Post-traumatic growth, Resilience

## Abstract

**Background:**

This article aims to examine how psychological resilience influences the interplay between quality of life and post-traumatic growth among breast cancer patients receiving follow-up care and treatment in Türkiye.

**Methods:**

The study involved 119 female individuals diagnosed with breast cancer who visited the Oncology outpatient clinic at a state hospital in Türkiye from January to September 2023. Data were gathered through the administration of a survey form and the utilization of several assessment tools, including the Adult Life Quality Scale in Cancer Survivors (QLACS), the Brief Resilience Scale (BRS), and the Post-traumatic Growth Inventory (PTGI). Data analysis was carried out using SPSS 25 software.

**Results:**

The participants demonstrated an inverse correlation between Post-Traumatic Growth (PTG) and two QLACS sub-dimensions, namely recurrence and family concern. Conversely, a positive association was identified between PTG and the advantages of dealing with cancer. Furthermore, a statistically significant positive association was established between BRS and all QLACS sub-dimensions, except for family concern and appearance. However, it was determined that psychological resilience did not act as a moderator in the relationship between PTG and QLACS.

**Conclusion:**

It is important to enhance psychological resilience in women who have survived cancer at all stages of the cancer journey, including the years after treatment, to have a positive impact on post-traumatic growth and quality of life.

## Introduction

Breast cancer continues to be the most prevalent cancer type among women. Due to the increasing incidence of breast cancer, advancements have been made in the treatment of the disease, leading to improvements in disease diagnosis and treatment over time, resulting in extended survival periods for patients. Today, in addition to survival, the quality of life has become a significant outcome measure in clinical research and survival studies related to breast cancer [1]. While numerous countries have recognized the significance of supportive care and rehabilitation, breast cancer survivors in various regions are often discharged after concluding their treatment, lacking ongoing monitoring or assistance for potential adverse effects, except for routine recurrence examinations [2].

As the chances of survival improve with treatments, new challenges emerge, especially in the context of the quality of survival [3]. It is common for breast cancer survivors to experience physical and emotional difficulties during the treatment and recovery phases. These difficulties may pose a threat to their capacity to sustain autonomous lifestyles and may likewise diminish their quality of life related to health [4]. Individuals often experience anxiety, depression, fears of disease recurrence, sleep disturbances, as well as physical pain and fatigue problems [5]. Furthermore, individuals diagnosed with breast cancer may encounter alterations in their physical appearance due to the therapeutic interventions they undergo. Such alterations encompass skin discoloration, dermatitis, discomfort associated with radiotherapy, potential loss or distortion of one or both breasts, hair loss, which could be a consequence of chemotherapy, and the presence of surgical scars. In certain instances, chemotherapy and hormone therapy might lead to weight gain and premature onset of menopause [6]. In patients who have undergone mastectomy, issues such as a decrease in self-esteem, a perception of a threat to fertility, and a lack of feeling attractive may be observed [7]. All these factors negatively impact the quality of life of individuals.

Nonetheless, individuals who have overcome cancer may also encounter significant beneficial transformations. One such positive transformation is post-traumatic growth, a concept characterized as “the emergence of constructive change following the grappling with exceptionally demanding life crises” [8]. With this concept, it is suggested that individuals exposed to various types of trauma not only experience commonly reported negative symptoms but also have the capacity for growth in their lives [9]. Cancer, being a traumatic experience, can lead patients to search for new meanings in their lives due to its impact on their beliefs and goals [10]. In the existing literature, it has been noted that post-traumatic growth is a prevalent occurrence among women diagnosed with breast cancer. Furthermore, there is a rising tendency in post-traumatic growth among survivors of early stage breast cancer over a span of 6 months [8]. Furthermore, it has been found that post-traumatic growth in cancer survivors is associated with a better quality of life [11].

Post-traumatic growth is the phenomenon of experiencing positive psychological changes in the aftermath of navigating a traumatic event. One's personal resilience can act as a protective factor in this journey, and the degree of resilience also significantly influences the management of a cancer diagnosis [12]. Resilience is defined as an individual's capacity to effectively navigate challenges and adjust to them. This ability is influenced by various factors, including cognitive, emotional, behavioral, spiritual, and physical aspects, all of which are integral components of post-traumatic growth [13]. Despite the significant distress associated with a cancer diagnosis and treatment, many cancer patients demonstrate remarkable resilience [14]. Within the existing body of literature, it is evident that resilience plays a moderating role in the quality of life across various health conditions. For instance, in a study involving patients with primary glaucoma, it was observed that resilience had a partial moderating impact on the correlation between symptom experiences and quality of life [15]. In a research investigation involving hemodialysis patients, it was observed that psychological resilience exerted a partial moderating influence on the connection between symptom experiences and quality of life [16]. There is a gap in the existing literature regarding the influence of psychological resilience as a moderator on the quality of life and post-traumatic growth in breast cancer survivors.

This article aims to investigate the moderating function of resilience in the relationship between post-traumatic growth and quality of life among patients diagnosed with breast cancer who are undergoing follow-up and treatment.

## Methods

This research is of a descriptive cross-sectional type. The research took place at the Oncology Polyclinic of a training and research hospital from January to September 2023. The research population included female patients who sought treatment at this facility, received a breast cancer diagnosis, and fulfilled the study's inclusion criteria.

The Research Inclusion Criteria are as follows:

- Volunteering to participate in the research,
- Having been diagnosed with cancer for at least 3 years,
- Being informed about cancer diagnosis,
- Having completed the active treatment process (not receiving chemotherapy, radiotherapy, hormone therapy, or surgical treatment),
- Continuing routine follow-up checks,
- Being between the ages of 18–80
- No history of recurrence or metastasis,
- Not being in the terminal period,
- Having the ability to read, write, speak, and understand Turkish,
- Not having a physical and/or mental health problem that may prevent from answering questions effectively.

Criteria for Exclusion from the Research:

- Incomplete answers to research questions

The number of patients applying to the relevant unit within a year is 150 people. Therefore, to determine the sample size for the study, the calculation formula intended for situations where the population size is known was applied. In cases where the universe is known, the number of people to be reached with a margin of error of 0.05 is 108. The research was completed with the participation of 119 people.

The study employed a survey designed by the researcher, the Adult Quality of Life Scale in Cancer Survivors, the Brief Psychological Resilience Scale, and the Post-traumatic Growth Inventory.

*Survey Form:* This form was created by the researchers by reviewing the literature on the subject. It generally consists of questions related to socio-demographic characteristics, such as age, occupation, education level, as well as questions regarding the treatment received for cancer and the duration of diagnosis [2, 3, 7, 8].

*Adult Quality of Life Scale in Cancer Survivors (QLACS):* Taylan and colleagues adapted the scale into Turkish. The overall quality-of-life scale consists of 7 domains: cognitive and emotional problems, sexual problems, social avoidance, positive emotions, pain, and energy/fatigue. The study reported a Cronbach alpha value of 0.90 for the scale [17].

*Brief Resilience Scale (BRS)*: The scale, originally created by Smith and colleagues in 2008 [18], was culturally adapted to Turkish by Doğan [19]. This is a 5-point Likert-type measurement instrument comprising 6 items and a singular dimension. Assessment of the scale's internal consistency revealed a Cronbach Alpha coefficient of 0.83 [19].

*Post-traumatic Growth Inventory:* The Post-traumatic Growth Inventory is a 6-point Likert-type self-report scale consisting of 21 items, developed by Tedeschi and Calhoun [20] to measure the level of changes as a result of a traumatic experience. The sub-dimensions of the scale are “Relating to others”, “New possibilities”, “Personal strength”, “Spiritual change”, and “Appreciation of life”. While the scores regarding internal consistency were 0.90 in the original scale [21], they were found to be 0.88 in this study.

The data were analyzed using the SPSS 25 software package. Percentages and means were used for data evaluation, while group comparisons were made using Student’s t test, ANOVA, and Tukey test. To assess the relationships between variables, correlation analyses and regression analyses were conducted, and Hayes PROCESS Model 4 was employed for specific analyses [22].

## Results

### The average scores that participants received on the scales.

In this research, the average scores of patients on the QLACS scale subscales, respectively, are as follows: Cognitive and emotional problems 25.56 ± 8.95, sexual problems 12.26 ± 5.29, social avoidance 11.92 ± 5.81, positive emotions 17.51 ± 5.95, pain 13.99 ± 6.39, and fatigue 11.20 ± 3.98. The total summary score for the Generic domains of the scale is 89.43 ± 25.69. When we examine the cancer-specific quality of life scores on the scale, we find the following: distress recurrence 14.92 ± 6.62, financial problems 14.94 ± 6.60, family distress 13.88 ± 5.37, appearance 13.15 ± 6, benefits 19.79 ± 4.75, and the summary score for this section (excluding benefits) is 56.91 ± 18.77.

The Brief Resilience Scale score is 19.7 ± 4.75. The average total score for PTG is 58.94 ± 21.84. The values of the scale’s sub-dimensions are as follows: “relating to others” 18.89 ± 7.68, “new possibilities” 14.35 ± 5.55, “personal strength” 11.15 ± 4.72, “spiritual change” 5.96 ± 5.55, and “appreciation of life” 5.90 ± 2.54 (Table 1).

**Table 1:** Descriptive statistics of participations’ Adult Life Quality Scale in Cancer Survivors, Brief Resilience Scale, and Post-traumatic Growth Inventory (n = 119)

Scales	Mean ± SD
Adult life quality scale generic domains	
Cognitive and emotional problems	25.56 ± 8.95
Sexual problems	12.26 ± 5.29
Social avoidance	11.92 ± 5.81
Positive emotions	14.48 ± 5.95
Pain	13.99 ± 6.39
Fatigue	11.20 ± 3.98
Summary score	89.43 ± 25.69
Cancer-specific domains	
Distress recurrence	14.92 ± 6.60
Financial problems	14.94 ± 6.60
Family distress	13.88 ± 5.37
Appearance	13.15 ± 6.0
Summary score*	56.91 ± 18.77
Benefits	17.87 ± 6.22
Brief resilience scale	19.79 ± 4.75
The Post-traumatic Growth Inventory (PTG)	58.94 ± 21.84
Relating to others	18.89 ± 7.68
New possibilities	14.35 ± 5.55
Personal strength	11.15 ± 4.72
Spiritual change	5.96 ± 5.55
Appreciation of life	5.90 ± 2.54

SD: standard deviation

^*^Benefits not included in summary score

### The correlations between post-traumatic growth, quality of life, and psychological resilience

Table 2 shows the correlations between the scales. It has been determined that there is a negative correlation between PTG and recurrence concern (*r* = − 0.204, *p* = 0.026) and family concern (*r* = − 0.205, *p* = 0.025), and a positive and statistically significant relationship between PTG and the benefit of cancer (*r* = 0.486, *p* = 0.000). There was also a negative correlation found between the score of relating to others and recurrence concern (*r* − 0.220, *p* = 0.016), and a positive and statistically significant relationship with the benefit of cancer (*r* = 0.458, *p* = 0.000). A positive correlation was found between the score of new opportunities and social avoidance (*r* = 0.190, *p* = 0.038), positive emotions (*r* = 0.234, *p* = 0.010), and the benefits of cancer (*r* = 0.557, *p* = 0.000). On the other hand, there was a statistically significant negative relationship between family concern and the score of new opportunities (*r* = − 0.209, *p* = 0.023). There is a negative relationship between personal resilience score and family anxiety (*r* = − 0.235, *p* = 0.010). A positive, statistically significant relationship was found between the personal strength score and the benefits of cancer (*r* = 0.500, *p* = 0.000). A negative relationship (*r* = − 0.186, *p* = 0.043) was determined between spiritual change and cognitive and emotional problems. A positive relationship (*r* = 0.338, *p* = 0.000) was determined between appreciation of life and the benefit of cancer.

**Table 2:** The correlations between post-traumatic growth, quality of life, and psychological resilience

		Cognitive and emotional problems	Sexual problems	Social avoidance	Positive emotions	Pain	Fatigue	Distress recurrence	Financial problems	Family distress	Appearance	Benefits
PTG total score	*r*	**− **0.129	0.006	0.096	0.159	**− **0.116	**− **0.085	− 0.204*	**− **0.074	− 0.205*	**− **0.126	0.486**
Relating to others	*r*	**− **0.106	0.001	0.122	0.131	**− **0.109	**− **0.049	− 0.220*	**− **0.057	**− **0.169	0.097	0.458*
New possibilities	*r*	0.007	0.096	0.190*	0.234*	**− **0.019	0.040	**− **0.123	0.006	− 0.209*	**− **0.094	0.557**
Personal strength	*r*	**− **0.094	0.022	0.057	0.129	**− **0.136	**− **0.069	**− **0.156	**− **0.107	− 0.235*	**− **0.167	0.500**
Spiritual change	*r*	− 0.186*	**− **0.052	**− **0.061	0.059	**− **0.087	**− **0.155	**− **0.133	**− **0.108	**− **0.120	**− **0.045	0.114
Appreciation of life	*r*	**− **0.076	**− **0.023	0.134	0.050	**− **0.082	**− **0.069	**− **0.043	0.051	**− **0.029	**− **0.085	0.338**
BRS	*r*	0.385**	0.255**	0.253**	0.458**	0.279**	0.290*	0.318**	0.225*	**− **0.013	0.067	0.333**

*Correlation is significant at the 0.05 level (2-tailed)

**Correlation is significant at the 0.01 level (2-tailed)

A statistically significant positive relationship was found between BRS and all dimensions of QLACS except family distress and appearance.

### The moderating role of psychological resilience in the impact of post-traumatic growth on quality of life

The results of the regression analysis aimed at determining the moderating role of psychological resilience in the impact of post-traumatic growth on cancer-specific quality of life are presented in Table 3. According to the analysis results, post-traumatic growth significantly and positively influences psychological resilience (*b* = 0.018, SE = 0.019, *p* < 0.001). Post-traumatic growth explains 4% of the variance in the psychological resilience variable. This result indicates that as post-traumatic growth increases, psychological resilience also increases (path a).

**Table 3:** The moderating role of psychological resilience in the impact of post-traumatic growth on quality of life

		Resulting variables								
		BRS (mediator)				QLACS (variable Y) (cancer-specific summary score)				
		β	SE	%95 BCA confidence interval			β	SE	%95 BCA confidence interval	
				LLCI ULCI					LLCI	ULCI
PTG (variable X)	Path a	0.018	0.019	− 0.0213	0.0573	Path c’	− 0.187	0.076	− 0.3388	− 0.0368
	*R*^2^ = 0.040, *p* = 0.3656									
BRS		–	–	–	–	Path b	0.929	0.354	0.2274	1.6297
Constant										
							*R*^2^ = 0.093, *p* = 0.000			
		Total effect X on Y				Path c	− 0.1710	0.0778	− 0.3252	− 0.0169
		Direct effect X on Y				Path c’	− 0.1878	0.0762	− 0.3388	− 0.0368
		Indirect effect X on Y				a.b	0.0167	0.012	− 0.0351	0.0703

*R*^2^: Adjusted R square; B: Partial regression coefficient; β: Standard partial regression coefficient; 95% CI: 95% confidence interval

**Fig. 1:**
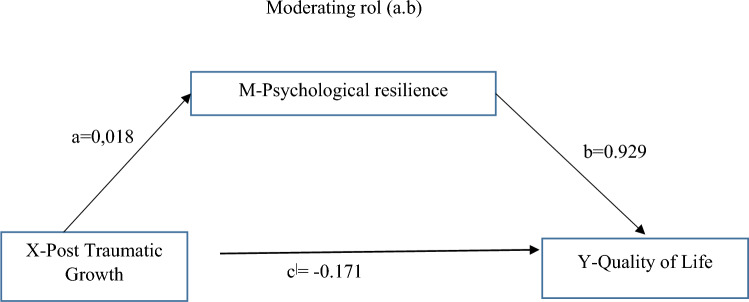
Conceptual model of moderation effect

The regression analysis has shown that post-traumatic growth has a significant and negative effect on the total score of cancer-specific quality of life (*b* = − 0.187, SE = 0.076, *p* < 0.01). Thus, as post-traumatic growth increases, cancer-specific quality of life decreases (path b). When post-traumatic growth is controlled, it was found that the direct effect of psychological resilience on cancer-specific quality of life (path c’) is significant (*b* = − 0.1878, SE = 0.0762, *p* < 0.01) (Fig. 1).

In the final stage, the indirect effect of post-traumatic growth on cancer-specific quality of life through psychological resilience was examined using the bootstrapping method (a.b), and it was found that the indirect effect of post-traumatic growth on cancer-specific quality of life was not significant. Because, according to the bootstrap analysis, the corrected and accelerated 95% confidence interval (95% BCA CI) contains 0 (zero) [95% BCA CI (− 0.0351 0.0703)]. Trauma post-growth and psychological resilience explain approximately 9% of cancer-specific quality of life (*p* < 0.001).

## Discussion

Research in the literature has indicated that individuals diagnosed with cancer often experience a reduced quality of life [23]. Improving the quality of life in patients diagnosed with breast cancer should be one of the important objectives [24]. This study aimed to examine the moderating effect of psychological resilience in the relationship between post-traumatic growth and quality of life in patients diagnosed with breast cancer.

In our study, we observed a negative relationship between post-traumatic growth and recurrence concerns, whereas a significant positive relationship was found between post-traumatic growth and the benefits of cancer. Therefore, as post-traumatic growth increases, individuals' recurrence concerns decrease, and their cancer benefit scores increase. Darabos and colleagues did not find a significant relationship between the fear of cancer recurrence and PTG [25]. Cancer survivors may begin to report positive outcomes of their illness as a result of coping with the challenging process. For instance, it has been noted that women diagnosed with breast cancer may report improved personal relationships, changes in priorities, and an increased appreciation for life after their diagnosis, which can be perceived as benefits arising from their illness [26]. Liu et al. found that the stage of cancer, recurrence of the disease, and the time since diagnosis were associated with the benefit of cancer and post-traumatic growth [11]. In a study conducted with patients who underwent breast cancer surgery, it was found that within 24 months after the surgery, there is a significant positive relationship between post-traumatic growth and physical and mental quality of life [27]. In a study conducted in Ghana, a statistically significant positive relationship was found between post-traumatic growth (PTG) and quality of life in individuals diagnosed with breast cancer [28]. In a comprehensive review study that examined the psychosocial determinants of quality of life in breast cancer survivors, it was noted that there is an association between post-traumatic growth and quality of life [29]. Post-traumatic growth and increasing resilience are important elements in the care of individuals with cancer [14]. Li et al. determined a positive relationship between post-traumatic growth and quality of life in patients [30]. The phenomenon of post-traumatic growth, which involves valuing life more after a traumatic event, is expected to result in individuals who also have the highest scores in positive emotions and the benefits of cancer having a better quality of life [31]. Our study has also yielded results that align with the literature (Fig. 1).

Psychological, physical, and social factors significantly affect the quality of life of individuals with cancer [32]. In this study, a positive, statistically significant relationship was determined between psychological resilience and all dimensions except family anxiety and appearance, which are sub-dimensions of the quality-of-life scale. Mohlin et al. found that psychological resilience was significantly related to all areas of the quality of life scale in individuals diagnosed with breast cancer [33]. Gündoğmuş et al. discovered a positive correlation between psychological resilience and post-traumatic growth in female breast cancer patients [34]. In addition, Boskailo et al. did not find a statistically significant relationship between psychological resilience and quality of life in patients diagnosed with breast cancer [35]. In a study, psychological resilience was associated with post-traumatic growth [36]. Michalczyk et al. found a positive relationship between psychological resilience and post-traumatic growth in women diagnosed with breast cancer [37]. Considering the positive outcomes of interventions aimed at increasing resilience in the literature, it is recommended that interventions to enhance resilience be urgently planned and implemented, especially for individuals with high post-traumatic stress and those adversely affected psychologically. Activities aimed at strengthening resilience can help mitigate the negative impact of physical, psychological, and social changes that occur during treatment [37]. In this study, it was determined that psychological resilience does not play an indirect moderating role in the relationship between post-traumatic growth and quality of life. In the literature, resilience is suggested to serve as a protective factor related to the use of active coping strategies and may moderate its relationship with quality of life [12]. Zhou et al. identified resilience as an important moderator among coping styles, perceived social support, and quality of life in their study [38]. A study involving individuals diagnosed with breast cancer suggested that psychological resilience could play a role in maintaining a balance between anxiety, depression, and post-traumatic growth [13]. In a study conducted with colorectal cancer patients, it was determined that psychological resilience was positively correlated with post-traumatic growth, while self-perception burden was negatively correlated with post-traumatic growth, and psychological resilience played a moderating role [39]. Although psychological resilience did not play a moderating role in the relationship between PTG and QOL in this study, it is believed that intervention studies aimed at increasing psychological resilience may have an impact on quality of life and post-traumatic growth. A nurse-led intervention study found increased post-traumatic growth and decreased anxiety and depression in breast cancer survivors [10]. Mindfulness-based stress coping training has been recommended as a complementary or adjunctive treatment for breast cancer patients [5]. Positive effects of cyclic adaptation training delivered via mobile device in post-operative breast cancer patients in China were determined in increasing psychological resilience and reducing symptoms of anxiety and depression [40]. Family and close circle support after treatment have a strong impact on increasing resilience and adapting to life after cancer [41]. It is thought that interventions aimed at increasing resilience, such as these and similar ones, may be beneficial for breast cancer survivors.

Limitations of this study include the fact that it was conducted in a single hospital, which limits the generalizability of the study results. Additionally, the fact that the entire sample consisted of female patients prevents us from understanding the situation in male patients.

## Conclusion

Our research has shown that the trauma caused by breast cancer can lead to positive changes in patients' lives, and that they experience post-traumatic growth. Post-traumatic growth decreases as the fear of recurrence increases. This indicates the need for us to address these concerns of patients. An increase in post-traumatic growth in patients is associated with an improvement in their quality of life and suggests that cancer can change their perspective on life.

It was determined that psychological resilience does not play a moderating role in the relationship between post-traumatic growth and quality of life.

## Data Availability

The data that support the findings of this study are available from the corresponding author upon reasonable request.
